# Lumbar zygapophyseal joints injections under ultrasound guidance an alternative to fluoroscopy guidance in the management of low back pain

**DOI:** 10.1038/s41598-022-07695-2

**Published:** 2022-03-07

**Authors:** Estelle Touboul, S. Salomon-Goëb, M. Boistelle, J. Sobhy Danial, V. Deprez, V. Goëb

**Affiliations:** Department of Rheumatology, Amiens University Hospital, 1 Place Victor Pauchet, 80000 Amiens, France

**Keywords:** Skeleton, Osteoarthritis, Chronic pain

## Abstract

Ultrasound-guided injections are an alternative to evaluate in the management of low back pain associated with osteoarthritis of the lumbar facet joints: it eliminates the risk of ionizing radiation for both the patient and the practitioner. This study aims to compare the short-term clinical efficacy of lumbar facet joints injections between ultrasound-guided injections and fluoroscopy-guided injections. Observational, retrospective, single-center study. Patients received one or two lumbar zygapophyseal joints injections under fluoroscopy or ultrasound and a follow-up consultation at one month. Data from 54 patients was collected. The median of the evolution of VAS at one month was − 30 mm [−50.0; −20.0] in the ultrasound group and – 29.5 mm [−47.0; −15.0] (p < 0.001) in the fluoroscopy group with no statistically significant difference between the two groups (p = 0.835). There were no injection-related complications in either group during the follow-up. The percentage of patients who stopped NSAIDs was not statistically significant between the two groups (p = 1.00). Injections of corticosteroids of lumbar facet joints under ultrasound guidance significantly reduced pain after one month, with no difference found between the two techniques. Ultrasound-guided injections are reliable, accessible and a safe alternative that should be preferred over those under fluoroscopy.

## Introduction

The degeneration of lumbar zygapophyseal joints can be responsible for low back pain. Radio-guided intra-articular injections have been suggested using a local anesthetic in combination with corticosteroids since 1976^1^. Intra-articular corticosteroid injections are used when lumbar facet syndrome is disabling and resistant to a well-conducted medical treatment as well as to hygiene and dietetic rules^2^. Commonly, the injections are done under scopic spotting allowing intra-articular position of the needle, according to international recommendations for spinal infiltration^3^. However, there is the question of the risks caused by ionizing radiation, especially since there is little data on the radiation protection of patients and caregivers. A 2016 French study found that injections of lumbar zygapophyseal joints are the most irradiating intervention under fluoroscopy in rheumatology, with a median dose of 175 microgray/m^2^ (76.4–180.1) for the infiltration of four lumbar zygapophyseal joints^4^. Ultrasound is an imaging technique that is more and more used by rheumatologists^5^. It is a diagnostic tool in addition to the clinical examination in pathologies of the musculoskeletal system, used for example in inflammatory rheumatism as rheumatoid arthritis and gout^6^ but also in mechanical pathologies as sarcopenia or exploration of the shoulder rotator cuff^7^. Interventional ultrasound in rheumatology has experienced considerable momentum in recent years, therefore infiltrations under ultrasound guidance are now the daily life of the rheumatologist^8^. The advantages of interventional ultrasound are the absence of irradiation, dynamic guidance, visualization of neighbouring structures (vessels and nerves) and therefore reduction of the traumatic risk, visualization of the injection of the potential products (deposits of corticosteroid crystals for example). The drawbacks are minimal, they are represented by the conditions specific to carrying out any infiltration.

The first study on the feasibility of lumbar zygapophyseal joints infiltrations under ultrasound guidance was performed in 1997 on 78 patients^9^; then several studies have confirmed and subsequently described the feasibility of this technique^10,11^. According to a meta-analysis carried out in 2016 only three studies (with a total of 202 patients) investigated the effectiveness of identifying lumbar zygapophyseal joints with ultrasound in comparison with scopic spotting that is still the preferred technique in use today^12^. Therefore, it seems interesting to study a comparable alternative in terms of efficacy and tolerance to infiltrations under fluoroscopy, accessible to any rheumatologist trained in osteoarticular ultrasound.

The main objective of this study was to compare the short-term efficacy of injections of lumbar facet joints in the management of low back pain between ultrasound-guided injections and fluoroscopy-guided injections.

## Method

This was an observational, retrospective, single-center study carried out at the University Hospital of Amiens in the Department of Rheumatology. The study period is between November 2018 and January 2020.

The study was approved by the CNIL (National Commission for Informatics and Freedom) registration number of the research project is the following PI2021_843_0031. All methods were carried out in accordance with relevant guidelines and regulations. Informed consent was obtained from all subjects.

The inclusion criteria were the following: age > 18 years, chronic lumbar pain with facet syndrome diagnosis by the rheumatologist, patients having received from one or two injections of lumbar zygapophyseal joints under fluoroscopy or under ultrasound in the Department of Rheumatology at the University Hospital of Amiens from November 2018 to January 2020, patients who have benefited from a one-month follow-up consultation.

The indication had been set during a consultation with a rheumatologist after diagnosis of a disabling clinical and radiographic lumbar facet syndrome, despite optimal and appropriate medical treatment. Patients answered the various quality of life questionnaires during the initial consultation and the follow-up consultation (EIFFEL^13^, OWESTRY^14^ and HAQ^15^).

### Injection under scopic guidance

**Figure 1:**
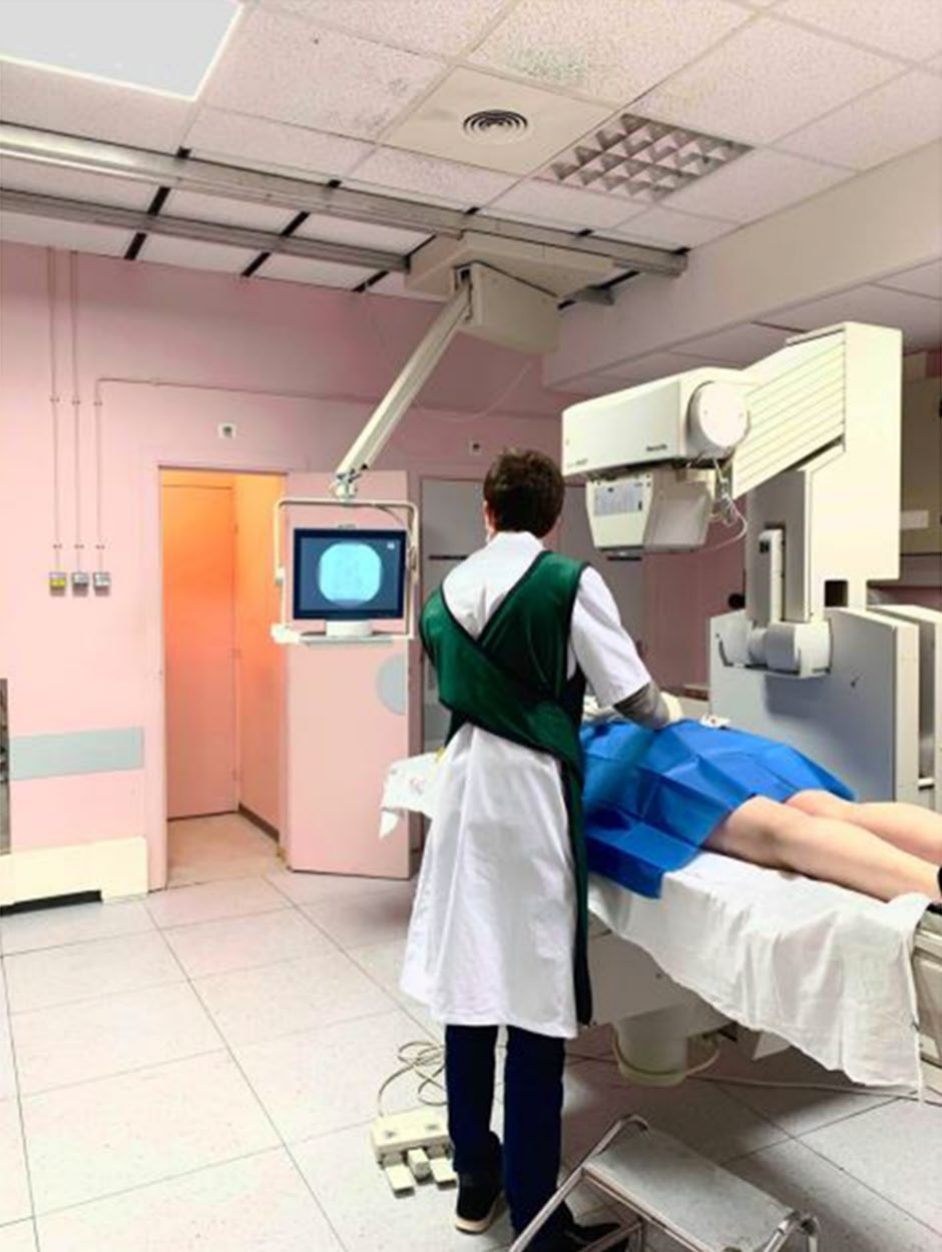
Injection of the right lumbar facet joint L4–L5 under fluoroscopy.

**Figure 2:**
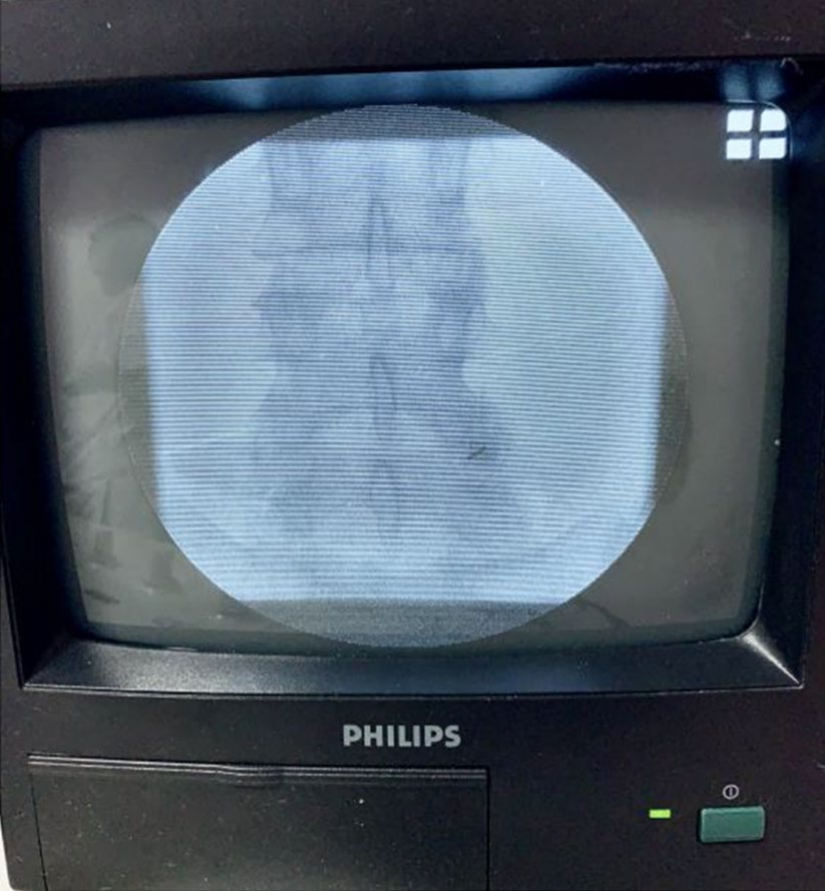
Injection of the right lumbar facet joint L4–L5 under fluoroscopy.

The injections were performed under scopic spotting in a dedicated radiology room and carried out under strict asepsis rules. Lumbar zygapophyseal joints were identified by fluoroscopy, then the needle lumbar puncture (20G) was introduced perpendicularly and advanced gently until bone contact with positioning and adaptation of the needle throughout the procedure under fluoroscopic control. Each symptomatic joint was injected with 1 ml of betamethasone. The duration of the intervention (from disinfection to removal of the needle) was recorded, as well as the number de Gray received, the immediate post-gesture complications and finally the pain VAS experienced by the patient during the intervention (Figs. 1 and 2).

### Injection under ultrasound guidance

**Figure 3:**
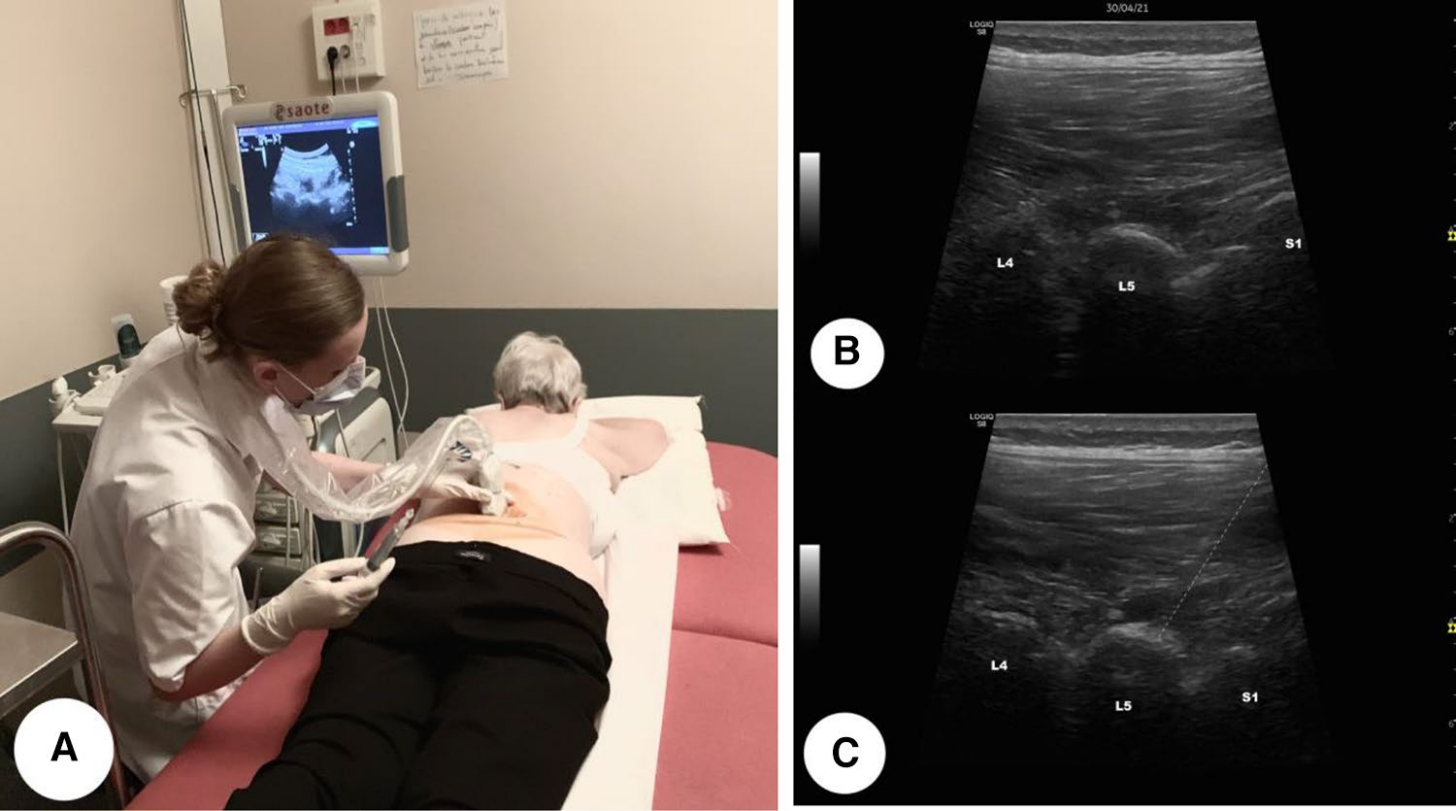
**(A)** Photograph showing the position before the injection of a lumbar facet joint L4–L5 under ultrasound-guidance. **(B)** Ultrasound before injection: we visualize several “bumps” that represent lumbar facet joints L4–L5 and L5–S1. **(C)** Ultrasound during injection: the dashed line in picture represents the needle path.

Ultrasound is performed with a low frequency, convex probe (1–8 MHz). We used a tracking in mode B in two perpendicular planes, to locate the target. The probe was positioned vertically approximately 3 to 4 cm to the left or to the right of the line of the spinous processes, the ultrasound appearance is easily spotted, as they form several “bumps”. The injection was carried out under strict aseptic conditions, the lumbar puncture needle (20G) was introduced into the axis of the probe with an angle of approximately 45°. The needle was advanced using local tracing anaesthesia using about 5 cc of Xylocaine 5% until bone contact, with positioning and adaptation of the needle throughout the procedure dynamically. Each symptomatic joint was injected with 1 ml of betamethasone^10^. The duration of the intervention (from disinfection until removal of the needle) was recorded, as well as the immediate post-gesture complications and finally the pain VAS felt by the patient during the operation (Fig. 3).

### Evaluation of the primary endpoint and secondary endpoints

During a check-up post-gesture consultation at about one month, the following were collected: the pain VAS and the various quality of life questionnaires (EIFFEL, OWESTRY, HAQ). We also collected information on complications and side effects reported by the patient after one month. Other patient data were collected including their medical history: inflammatory rheumatism included rheumatoid arthritis, spondyloarthritis, psoriatic arthritis, articular lupus, psychiatric history, heavy co-morbidities represented by at least two pathologies in the cardiovascular fields or oncology fields. The data on physiotherapy corresponded to the patient's declaration of having performed physiotherapy sessions prior to carrying out injections (10 sessions minimum).

### Statistics

Continuous variables were reported as median [interquartile 25–75%] and categorical variables as numbers and percentages (percentages were calculated excluding missing data). For exploratory purposes, comparison between the two methods of lumbar facet joint injections (ultrasound guidance versus fluoroscopic guidance) were performed using Wilcoxon–Mann–Whitney test for continuous variables and Chi2 test or Fisher’s exact test for qualitative variables. Change between M0 and M1 in pain and various scores (EIFEL, OWESTRY, HAQ) were described and compared using a Wilcoxon Signed Rank test. For patients who have undergone lumbar facet joint injections under fluoroscopic guidance, correlation between body mass index and radiation dose received were assessed using Spearman correlation coefficient and 95% confidence interval.

For all analyses, P < 0.05 was considered statistically significant. Because of the exploratory character of the comparisons, the type I error was not adjusted for multiplicity. All statistical analyses involved use of SAS 9.4 (SAS Inst. Inc, Cary, NC).

## Results

Three hundred seventy-one patients underwent one or two injections of lumbar zygapophyseal joints in the Department of Rheumatology at the University Hospital of Amiens from November 2018 to January 2020. Fifty-four patients benefited of a one-month follow-up consultation. Data from fifty-four patients were collected, twenty-four patients received injections under ultrasound guidance and thirty patients under fluoroscopic guidance, corresponding to a total of ninety-eight lumbar facet joints.

### Patient characteristics

There was no statistically significant difference between the two groups for age, sex and BMI, medical history and the declared practice of physiotherapy (Table 1).

**Table 1:** Patient characteristics.

	Ultrasound (N = 24)	Scopy (N = 30)	Total (N = 54)	p intergroup comparison
**Sex**				0.890 (C)
N	24	30	54	
Women	18 (75.0%)	22 (73.3%)	40 (74.1%)	
**Age (years)**				0.965 (W)
N	24	30	54	
Median	66.0	63.5	64.5	
Q1–Q3	52.0–72.5	54.0–75.0	53.0–73.0	
**BMI (kg/m**^**2**^**)**				0.102 (W)
N	24	30	54	
Médian	25.9	28.5	27.4	
Q1–Q3	23.8–29.7	25.0–34.6	24.0–33.3	
**Heavy comorbidities**				0.950 (C)
n	24	30	54	
Yes	9 (37.5%)	11 (36.7%)	20 (37.0%)	
**History of spine surgery**				1.000 (F)
n	24	30	54	
Yes	1 (4.2%)	2 (6.7%)	3 (5.6%)	
**Inflammatory rheumatism**				1.000 (F)
N	24	30	54	
Yes	4 (16.7%)	5 (16.7%)	9 (16.7%)	
**Psychiatric history**				0.930 (C)
N	24	29	53	
Yes	8 (33.3%)	10 (34.5%)	18 (34.0%)	
**Physiotherapy**				0.891 (C)
N	23	30	53	
Yes	18 (78.3%)	23 (76.7%)	41 (77.4%)	

(C) Chi-2 test.

(F) Fisher test.

(W) Wilcoxon test.

### Number of injections

In the ultrasound group : six patients received one injection (25%), eighteen patients received two injections (75%). In the fluoroscopy group : four patients received one injection (13.3%) and twenty-six patients received two injections (86.7%). There was no statistically significant difference between the two groups in terms of number of injections (p = 0.311).

### Injection completion time

The injection time in total was collected in seconds and the time per lumbar zygapophyseal joint was calculated. The median was 91 s [70.0–109.5] per lumbar zygapophyseal joint in the fluoroscopy group and 126 s [84.0–187.5] in the ultrasound group (p = 0.007). There was a statistically significant difference between the two groups, with a longer time recorded for the ultrasound group.

### Radiation received by lumbar facet joint in the fluoroscopy group

The median radiation received per lumbar zygapophyseal joint was 47 µGy/m^2^ [24.7–60.0]. The coefficient of Spearman's correlation (non-parametric) between BMI and the radiation dose received was 0.544 with an interval 95% confidence [0.206–0.766].

### VAS infiltration pain

The pain VAS felt while performing the injection was collected in both groups. The median in the ultrasound group was 22 mm [0.0–45.0] and 30 mm [20.0–50.0] in the fluoroscopy group. The difference was not statistically significant between the two groups. (p = 0.271).

### Side effects of the injection

No immediate side effects after completion of the injection were observed in either group. No complications were reported by the patients during the follow-up consultation at 1 month.

### Assessment of the primary endpoint

The median of the pain VAS before performing the injection was 76.5 mm [65.0; 85.0] in the ultrasound group and 70.5 mm [62.0; 82.0] in the fluoroscopy group. The median of the evolution of pain at one month after injection in the ultrasound group was – 30.0 mm [−50.0; −20.0] (p < 0.001). The median of the evolution in pain at one month after injection in the fluoroscopy group was − 29.5 mm [−47.0; −15.0] (p < 0.001). There is a statistically significant decrease in pain VAS in both groups. There was no statistically significant difference in the evolution of the pain VAS at 1 month between the two groups (Table 2).

**Table 2:** VAS evolution at 1 month.

	Ultrasound (N = 24)	Scopy (N = 30)	Total (N = 54)	p intergroup comparison
**VAS M0**				0.397 (W)
n	24	30	54	
Median	76.5	70.5	75.0	
Q1–Q3	65.0–85.0	62.0–82.0	65.0–85.0	
**VAS M1**				0.602 (W)
n	23	28	51	
Median	40.0	40.0	40.0	
Q1–Q3	30.0–62.0	27.5–57.5	29.0–60.0	
**Evolution VAS M1–M0**				0.835 (W)
N	23	28	51	
Median	−30.0	−29.5	−30.0	
Q1–Q3	−50.0 to −20.0	−47.0 to −15.0	−48.0 to −18.0	
p value intra-group comparison	< 0.001 (RS)	< 0.001 (RS)	< 0.001 (RS)	

(RS) Signed rank test.

(W) Wilcoxon test.

### Evolution of drug consumption

Regarding NSAIDs, in patients taking NSAIDs before the injection: five out of eight patients stopped their consumption of NSAIDs in the ultrasound group, three out of four stopped their consumption in the fluoroscopy group. There was no statistically significant difference between both groups (p = 1.00). Regarding analgesic treatments other than NSAIDs in patients taking analgesic treatments before injection: eleven out of eighteen patients reduced their consumption in the ultrasound group, seven out of twenty-three patients reduced their consumption in the fluoroscopy group. There is a greater drop in analgesic consumption in the ultrasound group, but with no statistically significant difference between both groups (p = 0.05).

### Evolution of the EIFFEL/HAQ/OWESTRY score

There was a statistically significant improvement in the EIFFEL score in the two groups at one month after the infiltration, without a statistical significant difference found between the two groups (p = 0.233). There was a statically significant improvement in OWESTRY score in both groups at 1 month of infiltration, with no statistically significant difference found between the two groups. There was no significant difference in the evaluation of HAQ in the two groups, the median is −2.0 in the ultrasound group (p = 0.180) and −2.0 in the fluoroscopy group (p = 0.09). There was no statistically significant difference between the two groups (Table 3).

**Table 3:** Evolution Eiffel score, HAQ and OWESTRY at 1 month.

	Ultrasound (N = 24)	Scopy (N = 30)	Total (N = 54)	p intergroup comparison
**Evolution score EIFEL M1–M0**				0.233 (W)
n	23	24	47	
Median	−2.0	−1.5	−2.0	
Q1–Q3	−3.0 to 0.0	−7.0 to −1.0	−4.0 to 0.0	
p-value intra-group comparison	0.041	< 0.001	< 0.001	
**Evolution score HAQ M1–M0**				0.472 (W)
N	23	24	47	
Median	−0.1	−0.1	−0.1	
Q1–Q3	−0.3 to 0.1	−0.4 to 0.0	−0.4 to 0.0	
p-value intra-group comparison	0.180	0.009	0.004	
**Evolution score OWESTRY M1–M0**				0.137 (W)
N	23	23	46	
Median	−7.0	−10.0	−7.5	
Q1–Q3	−10.0 to 4.0	−14.0 to 0.0	−14.0 to 0.0	
p-value intra-group comparison	0.005	< 0.001	< 0.001	

(RS) Signed rank test.

(W) Wilcoxon test.

## Discussion

Our results showed a reduction in VAS pain at one month in both groups with a median decrease of – 30.0 mm [−50.0; −20.0] in the ultrasound group and – 29.5 mm [−47.0; −15.0] in the fluoroscopy group with no statistically significant difference. This is consistent with the data found in the literature on the reduction of VAS pain and the absence of difference from fluoroscopic infiltration^10,16,17^.

The action of corticosteroids is linked to their anti-edema, anti-inflammatory and pain-blocking effect transmitted by C fibers. In osteoarthritis disease, there is a local inflammatory reaction with the release of inflammatory mediators^18^ and therefore the corticosteroid injection can reduce this local inflammatory reaction resulting in a reduction of pain in short and medium term^19^. It is difficult to confirm in clinical practice that low back pain is strictly due to a pathology of lumbar zygapophyseal joints, which may explain the complete lack of analgesic effect in some patients.

Regarding the injection time, there was a statistically significant difference between the two groups, with a longer procedure in the ultrasound group than in the scopy group. The time to perform the fluoroscopic injection was very fast, with a median of 91 s [−70; 109.5] per facet joint. Our results are comparable with those found in the literature^16^. However, fluoroscopic procedures require the intervention of a radiology technician, which can increase the overall support time, the cost of implementation and decrease accessibility.

In the fluoroscopy group, the median radiation received by lumbar facet joint was 47 µGy/m^2^ [24.7; 69.0]. These results support the data reported in the literature, with a median dose of 43.75 µGy/m^2^ for a lumbar zygapophyseal joint^20^. This data confirm our interest in developing a non-irradiating alternative to this procedure, which is described as the most irradiating infiltration in interventional rheumatology. In addition, we wanted to study the correlation between BMI and the radiation dose received, and we showed that an increase of the BMI was correlated with an increase in the dose received (Spearman's correlation coefficient was 0.544). This information is also found in the literature reporting an increase in irradiation in patients with high BMI, especially since the procedure is technically more difficult and the detection longer in these patients^4^.

Regarding the pain felt during the procedure, the injection under ultrasound control was less painful than under fluoroscopy with a median VAS at 22 mm [20.0; 50.0] versus 30 mm [0.0; 45.0] in the ultrasound group. This difference was not statistically significant (p = 0.271). Visualization of the needle path and injection of Xylocaine as the needle progresses can likely explain this finding.

As found in the literature^10,16^, no side effects were reported (immediate side effects or one month after the injection)*.*

Our results showed an improvement in functional disability linked to low back pain (EIFFEL score) in both groups, with a median decrease of 2 points [−3.0; 0.0] in the ultrasound group and 1.5 [−7.0; −1.0] points in the fluoroscopy group, with no statistically significant difference. There is an improvement, moderate but real, in the functional abilities of patients in the short term. The OWESTRY score (an index to quantify the disability related to low back pain) in our study was slightly improved after infiltration under scopy and under ultrasound, with no statistically significant difference between the two groups. Concerning the HAQ functional score, there was no significant difference in the evolution of scores in the two groups. This lack of statistical difference can be explained by the small sample of patients in our study, but for some patients there may be a clinical benefit of these injections.

Our study was also interested in the evolution of consumption of analgesics after receiving the injection. It is interesting to note that there was a clinically significant decrease in the intake of NSAID drugs in the two groups, with no statistically significant difference found between both groups.

The present study had some limitations, only a small group of patients was included, which is explained by the fact that only few patients received a follow-up consultation at one month. It should be noted that infiltrations are carried out under radiographic identification (ultrasound or fluoroscopy) and that there were no infiltration carried out under anatomical identification as required by the current guidelines^21,22^. The absence of a control group (with injection of placebo instead of cortisone) and the absence of randomization does not allow us to conclude that the injections are effective. Finally, our study was carried out in only one center, therefore there was a selection bias.

Our results are consistent with those found in the literature^23^ and clearly highlight the ease of performing ultrasound infiltration, it’s accessibility and increased safety^12,24^. We have seen a small amount of data in the literature on the study of the effectiveness of injection under ultrasound guidance^12^ despite using this technique for numerous infiltrations in current practice^21^. Thus, our study brings some information on the practical execution of this injection in real life conditions, with the major advantage that this type of injection can be carried out in rheumatology practice, thus improving access to care for patients. This guide way ensures good reliability, reproducibility, and local safety while reducing the risk of radiation for the patient and the practitioner. Our data therefore reinforce the current trend in the development of ultrasound in musculoskeletal disease as a diagnostic tool, a pillar in interventional rheumatology and an aid in patient follow-up^5,6,11,25^.

## Conclusion

It appears that there is no difference in short-term efficacy between infiltration of lumbar facet joints under ultrasound versus scopic guidance. Injections under ultrasound guidance are a very interesting alternative to fluoroscopic guidance, they are reliable, accessible, and they overcome the risk of ionizing radiation for the patient and the practitioner.
